# Supplementary material for: Urine metabolomics of rats with chronic atrophic gastritis
Source: PLoS One. 2020 Nov 11;15(11):e0236203. doi: 10.1371/journal.pone.0236203 (PMC7657567; doi:10.1371/journal.pone.0236203)
Supplement: Attachment — Submitted filename: Response to Reviewers.docx [file pone.0236203.s002.docx]

Additional Editor Comments:Metabonomics research directions include genetic environment causes many factors, such as synthetic drugs' effects on the body of the system response, the research focus of metabonomics is medicine application is very extensive, widely applied in modern research of traditional Chinese medicine diagnosis drug toxicity evaluation of drug research and development, etc., such as researching the mechanism of action of traditional Chinese medicine (TCM) targets of traditional Chinese medicine syndrome meridian medical reports and safety evaluation of traditional Chinese medicine research field at present most of the potential biomarkers from urine, urine is not only collect invasive, but because in the urine urine rich and need a minimum of preparationTherefore, urine has been the most studied in demonstrating metabolic differences in various common and specific diseases, and the technology is now mature and has achieved good research results.

Journal Requirements:

1. The manuscript was revised in the style of PLOS ONE.
2. Before sampling, they fasted for 24 hours, drank normal water, collected urine, followed by anaesthesia with 2% pentobarbital sodium, blood collection of spleen, stomach and liver, and subsequent death.
3. Urine was collected and then used for further research.
4. Example ethics statement

This study was carried out in strict accordance with the recommendations in the Guidelines for ethical review of experimental Animal welfare(National Standard:GB/T 35892—2018). The protocol was approved by the Ethics Review Center of Shandong University of Traditional Chinese Medicine (Protocol Number:SDUTCM20190402003). All surgery was performed under sodium pentobarbital anesthesia, and all efforts were made to minimize suffering.

5. S3Fig and S6Fig

Fig. 3. Chromatogram in Total Ion Mode. 3-A: Typical Sample BPC in Positive Ion Mode, 3-B: Typical Sample BPC in Negative Ion Mode.

Fig. 6. Heat Map of the Differential Metabolites. A: Heat Map of the Differential Metabolites in CAG Rats; 6-B: Correlation Heat Map of Differential Metabolites in CAG Rats.

Reviewers' comments:

Reviewer's Responses to Questions

Comments to the Author.

The manuscript has been edited by American Journal Experts.

Reviewer #2:Urine test for diagnostic purposes: the necessity of early gastric cancer lacking characteristic symptoms, usually are diagnosed in the late, leading to poor prognosis and the prognosis of early gastric cancer was superior to that of advanced gastric cancer, so early diagnosis and treatment is the key to improving the prognosis of gastric cancer metabonomics as a new method in the field of systems biology, has become an integral part of the new tool in cancer research, and help to increase understanding of cancer pathogenesis and drug action mechanism of endogenous metabolic changes better understanding will promote the diagnosis and treatment of cancer in recent years, based on plasma urineOrganization and metabonomics analysis of gastric juice as well as the relationship between metabolic regulation and cancer research a series of progress has been made as biological specimens of detecting cancer biomarkers, urine is being more and more attention, not only because the urine is invasive collection, but also because in urine urine rich and need a minimum of preparation.

Rats are easier to get, and also easier to detect, because of their larger size and more pronounced symptoms.Compared to mice, rats are also economically viable.

The metabolic spectrum of rats can only provide a certain direction for clinical practice, but cannot provide complete guidance for clinical practice. These compounds can at least prove the success of the chronic atrophic gastritis model in rats, which is of certain significance for the diagnosis of diseases in rats.

Reviewer #2:

Specific comments:

1.PCA incredible figure outside the confidence interval for the abnormal samples, including format conversion in data processing before peak identification filter alignment and normalization of data pretreatment, its external data check plus or minus the total ion chromatograms of display and based on mass spectrometry of metabonomics research, in order to obtain reliable and high quality of metabolomics data, usually need to quality control, quality control (QC) .When testing using QC samples quality control theory, QC samples are the same, but in the process of sample extraction test analysis will be a system error, result in QC samples, there will be differences between the smaller the difference method of stability, the higher the better data quality, reflect on the PCA analysis diagram is the concentrated distribution of QC samples, specification data reliable QC samples gathered, good repeatability, stable system.1. In order to find biomarkers, the potential characteristic peak's relative standard deviation (RSD) in QC samples, that is, the coefficient of variation should not exceed 30%. If it does, the relevant characteristic peak should be deleted Assurance(QA) to remove the features with poor repeatability in QC samples in order to obtain a higher quality data set, which is more conducive to the detection of biomarkers in QC samples,RSD＜30%;The characteristic peak proportion can reach about 70%, indicating good data . Therefore, the data reflected in PCA confidence graph are valid data, while the samples outside the confidence interval are abnormal samples.PLS-DA and OPLS-DA: R2X, R2Y and Q2 relative to each model should be reported, as well as the number of wrong classifications in the training data set (i.e. internal validation).100 permutations have been chosen for models validation. Independent samples T-test should be used to determine in the different biomarker candidates obtained from the OPLS-DA models are statistically significant between the two groups at the univariate level.

2.In the registration of weight changes at 16 weeks, normality test was performed first, and then one-way ANOVA was used to conduct statistical test of weight between the blank group and the model group, and *P＜0.01 was found between the two groups,the difference was statistically significant.

3.LC-MS data is carried out on the basis of normalization, eliminating very few data that do not exist or have too low strength. The experiment adopts most data retained after QC, QA and normalization processing.Prior to urine metabolomics analysis, the Proteowizard software (V3.0.8789) was used to convert the obtained original data into mzXML format (XCMS input file format).Using R (v3.3.2) XCMS package is used to identify the peaks identification,peaks filtration,peaks alignment, the main parameters are bw = 5, PPM = 15, peakwidth = c (10, 20), mzwid = 0.015, mzdiff = 0.01, the method = centWave.The data matrix, including mass to charge ratio (M/Z), retention time (RT) and intensity, is obtained.In the positive ion mode 22,540 precursor molecules and the negative ion mode 18,837 precursor molecules were obtained. The data were exported to Excel for subsequent analysis. In order to make comparison of data of different orders, batch normalization of data regarding peak area was conducted.

4.The corrected positive ion data is after data pretreatment (format conversion peak recognition, filtering alignment and normalization), the data screened out have strong repeatability and good effect for urine metabolomics analysis

5.In the analysis of the diversity of urine metabolomics, potential biomarkers were identified through MS/MS experiments. Compounds were added in Table 1, including mass to charge ratio, M/Z Retention time, RT and Exact mass

6.In the * P＜0.5 and Impact＞0;The metabolic pathways of include D-glutamine and D-glutamate metabolizing,histamine and purine metabolizing pathways,In these pathways,D-glutamine and D-glutamic acid metabolism were up-regulated,three metabolites in histamine metabolism were down-regulated,and six metabolites in purine metabolism were down-regulated.

7.In recent years, metabonomics has been widely used in modern Chinese medicine treatment of chronic gastritis.Cui.Jiajia Et al.[8]found that 3 plasma biomarkers (arginine,succinate and 3-hydroxybutyrate) and 2 urine biomarkers (α-ketoglutarate and valine) might be markers of CAG in the study on plasma and urine metabolites.Chen jiaolong [9] by using nuclear magnetic metabonomics technology including observation of the stomach meridian the CAG treatment, use of electroacupuncture in the rat stomach meridians beam door and foot three mile, found that serum ghrelin level stomach metabolism of liver kidney brain cortex spectral change, the rat gastric mucosa arrangement and the thickness of the gastric mucosa has different degrees of improvement, ghrelin and substance P expression in serum increased to normal level, serum glucose glycogen content increased, the stomach tissue of glutathione and glutamine levels, hypoxanthine creatinine nicotinamide in brain tissueThe content of malonic acid and dimethyl malonate increased, the content of glucodicarbonate and glycerin in liver tissues increased, and the content of glutamine hypoxanthine nucleoside asparagine asparagine and nicotinamide in kidney tissues increased.Liu Caichun Et al.[10]found that both electroacupuncture and moxibustion could restore various caG-induced metabolic changes, including membrane metabolism, energy metabolism and neurotransmitter function.Liu Yuetao [11] based on 1 H-NMR technical analysis astragalus chienchung soup to the CAG rats serum endogenous metabolites regulation function disorder, through multivariate statistical analysis, to clarify its regulation on chronic atrophic gastritis targets, related indicators associated with efficacy is established, by partial least-squares regression analysis and Met PA screening and treatment effect is most related metabolic pathways, find the root of remembranous milk vetch chienchung soup can obviously inhibit the CAG lesions, can obviously regulate 3 - hydroxy butyric acid lactic acid acetate succinate metabolites such as disorder, the CAG treatment is the main metabolic pathways of arginine - proline metabolismGlycerol metabolism and glycine - serine - threonine metabolism pathways. Sun Yina [12] found 59 qualitative and quantitative metabolites, and then PCA OPLS-DA VIP value was used to find 8 potential differential metabolites related to dampis-heat syndrome of spleen and stomach. The metabolites of trimethyl-oxide of taurine gonosaccharide glycerol and glucose were up-regulated, and the metabolites of trimethyl-trimethyl-oxide and trigonelline phosphate creatine were down-regulated.It is concluded from the above studies that metabonomic techniques have been widely used in traditional Chinese medicine. However, 1H-NMR metabolism technology is mostly used in the studies on chronic atrophic gastritis. In this study, LC-MS technology is used to elucidate the small molecule action mechanism and related target pathways of CAG.

8.Some of the images resolution has been improved.
