# Supplementary material for: Urine metabolomics of rats with chronic atrophic gastritis
Source: PLoS One. 2020 Nov 11;15(11):e0236203. doi: 10.1371/journal.pone.0236203 (PMC7657567; doi:10.1371/journal.pone.0236203)
Supplement: Attachment — Submitted filename: PONE-D-20-19248_reviewer_2.docx [file pone.0236203.s003.docx]

| 1. PCA incredible figure outside the confidence interval for the abnormal samples,  including format conversion in data processing before peak identification filter  alignment and normalization of data pretreatment, its external data check plus or  minus the total ion chromatograms of display and based on mass spectrometry of  metabonomics research, in order to obtain reliable and high quality of metabolomics  data, usually need to quality control, quality control (QC) .When testing using QC  samples quality control theory, QC samples are the same, but in the process of sample  extraction test analysis will be a system error, result in QC samples, there will be  differences between the smaller the difference method of stability, the higher the  better data quality, reflect on the PCA analysis diagram is the concentrated  distribution of QC samples, specification data reliable QC samples gathered, good  repeatability, stable system.1. In order to find biomarkers, the potential characteristic  peak's relative standard deviation (RSD) in QC samples, that is, the coefficient of  variation should not exceed 30%. If it does, the relevant characteristic peak should be  deleted Assurance(QA) to remove the features with poor repeatability in QC samples  in order to obtain a higher quality data set, which is more conducive to the detection  of biomarkers in QC samples,RSD**＜**30%;The characteristic peak proportion can  reach about 70%, indicating good data . Therefore, the data reflected in PCA  confidence graph are valid data, while the samples outside the confidence interval are  abnormal samples.PLS-DA and OPLS-DA: R2X, R2Y and Q2 relative to each model  should be reported, as well as the number of wrong classifications in the training data  set (i.e. internal validation).100 permutations have been chosen for models validation.  Independent samples T-test should be used to determine in the different biomarker  candidates obtained from the OPLS-DA models are statistically significant between  the two groups at the univariate level. | - Which software have you used for statistical analysis (e.g. SIMCA)? - Univariate analysis should be conducted with p values adjusted for multiple testing. - Please add the information about permutation test in the text. |
| --- | --- |
| 2. In the registration of weight changes at 16 weeks, normality test was performed  first, and then one-way ANOVA was used to conduct statistical test of weight between  the blank group and the model group, and *P**＜**0.01 was found between the two  groups,the difference was statistically significant. | Please report this information in the text. |
| 3. LC-MS data is carried out on the basis of normalization, eliminating very few  data that do not exist or have too low strength. The experiment adopts most data  retained after QC, QA and normalization processing.Prior to urine metabolomics  analysis, the Proteowizard software (V3.0.8789) was used to convert the obtained  original data into mzXML format (XCMS input file format).Using R (v3.3.2) XCMS  package is used to identify the peaks identification,peaks filtration,peaks alignment,  the main parameters are bw = 5, PPM = 15, peakwidth = c (10, 20), mzwid = 0.015,  mzdiff = 0.01, the method = centWave.The data matrix, including mass to charge ratio  (M/Z), retention time (RT) and intensity, is obtained.In the positive ion mode 22,540  precursor molecules and the negative ion mode 18,837 precursor molecules were  obtained. The data were exported to Excel for subsequent analysis. In order to make  comparison of data of different orders, batch normalization of data regarding peak  area was conducted. | - Please report all these information in the text. - Which signal has been selected for peak normalization? Or do the authors mean that the signals have been normalized to the sum of peak areas? - Have been peak areas centered and/or scaled and/or transformed before multivariate analysis? |
| 4. The corrected positive ion data is after data pretreatment (format conversion  peak recognition, filtering alignment and normalization), the data screened out have  strong repeatability and good effect for urine metabolomics analysis | Ok |
| 5. In the analysis of the diversity of urine metabolomics, potential biomarkers  were identified through MS/MS experiments. Compounds were added in Table 1,  including mass to charge ratio, M/Z Retention time, RT and Exact mass | - Have xenobiotics been eliminated from the list of possible metabolites? As far as I know, compounds as marmesin acetate, syringic acid o taxifolin are not endogenous. |
| 6. In the * P **＜** 0.5 and Impact **＞** 0;The metabolic pathways of include  D-glutamine and D-glutamate metabolizing,histamine and purine metabolizing  pathways,In these pathways,D-glutamine and D-glutamic acid metabolism were  up-regulated,three metabolites in histamine metabolism were down-regulated,and six  metabolites in purine metabolism were down-regulated. | Ok |
| 7. .In recent years, metabonomics has been widely used in modern Chinese  medicine treatment of chronic gastritis.Cui.Jiajia Et al.[8]found that 3 plasma  biomarkers (arginine,succinate and 3-hydroxybutyrate) and 2 urine biomarkers (**α**  -ketoglutarate and valine) might be markers of CAG in the study on plasma and urine  metabolites.Chen jiaolong [9] by using nuclear magnetic metabonomics technology  including observation of the stomach meridian the CAG treatment, use of  electroacupuncture in the rat stomach meridians beam door and foot three mile, found  that serum ghrelin level stomach metabolism of liver kidney brain cortex spectral  change, the rat gastric mucosa arrangement and the thickness of the gastric mucosa  has different degrees of improvement, ghrelin and substance P expression in serum  increased to normal level, serum glucose glycogen content increased, the stomach  tissue of glutathione and glutamine levels, hypoxanthine creatinine nicotinamide in  brain tissueThe content of malonic acid and dimethyl malonate increased, the content  of glucodicarbonate and glycerin in liver tissues increased, and the content of  glutamine hypoxanthine nucleoside asparagine asparagine and nicotinamide in kidney  tissues increased.Liu Caichun Et al.[10]found that both electroacupuncture and  moxibustion could restore various caG-induced metabolic changes, including  membrane metabolism, energy metabolism and neurotransmitter function.Liu Yuetao  [11] based on 1 H-NMR technical analysis astragalus chienchung soup to the CAG  rats serum endogenous metabolites regulation function disorder, through multivariate  statistical analysis, to clarify its regulation on chronic atrophic gastritis targets, related  indicators associated with efficacy is established, by partial least-squares regression  analysis and Met PA screening and treatment effect is most related metabolic  pathways, find the root of remembranous milk vetch chienchung soup can obviously  inhibit the CAG lesions, can obviously regulate 3 - hydroxy butyric acid lactic acid  acetate succinate metabolites such as disorder, the CAG treatment is the main  metabolic pathways of arginine - proline metabolismGlycerol metabolism and glycine  - serine - threonine metabolism pathways. Sun Yina [12] found 59 qualitative and  quantitative metabolites, and then PCA OPLS-DA VIP value was used to find 8  potential differential metabolites related to dampis-heat syndrome of spleen and  stomach. The metabolites of trimethyl-oxide of taurine gonosaccharide glycerol and  glucose were up-regulated, and the metabolites of trimethyl-trimethyl-oxide and  trigonelline phosphate creatine were down-regulated.It is concluded from the above  studies that metabonomic techniques have been widely used in traditional Chinese  medicine. However, 1H-NMR metabolism technology is mostly used in the studies on  chronic atrophic gastritis. In this study, LC-MS technology is used to elucidate the  small molecule action mechanism and related target pathways of CAG. | Ok |
| 8. Some of the images resolution has been improved. | Ok |
