# Supplementary material for: Urine metabolomics of rats with chronic atrophic gastritis
Source: PLoS One. 2020 Nov 11;15(11):e0236203. doi: 10.1371/journal.pone.0236203 (PMC7657567; doi:10.1371/journal.pone.0236203)
Supplement: Attachment — Submitted filename: Response to Reviewers.doc [file pone.0236203.s004.doc]

Response to Reviewers

· Which software have you used for statistical analysis (e.g. SIMCA)?

RE：The analysis software used for multidimensional statistical analysis is SIMCA-P (V13.0).In addition, the calculation of P value is t-test

· Univariate analysis should be conducted with p values adjusted for multiple testing.

RE：The t-test that we use for univariate statistical analysis, in general,t-test is p＜0.05 is significant, p＜0.01 is very significant, biological statistical methods are basically such a display of differences;This section provides references for significance of biological repeated screening p values

· Please add the information about permutation test in the text.

RE：Permutation is the result of 200 permutation tests，PLD-DA permutation test in positive ion mode is R2=（0.0，0.91），Q2=（0.0，0）PLD-DA permutation test in negative ion mode is R2=（0.0，0.9），Q2=（0.0，-0.39）

· Which signal has been selected for peak normalization? Or do the authors mean that the signals have been normalized to the sum of peak areas?

RE：According to the results, the original peak area, which is the relative strength value, is calculated，then the original peak area is standardized,batch normalization of peak area of data.Data analysis is based on standardized data.

·Have been peak areas centered and/or scaled and/or transformed before multivariate analysis?

RE：According to the results, the original peak area, which is the relative strength value, is calculated，then the original peak area is standardized,batch normalization of peak area of data.Data analysis is based on standardized data.Multidimensional statistical analysis was performed based on the data after standardized processing

·Have xenobiotics been eliminated from the list of possible metabolites? As far as I know, compounds as marmesin acetate, syringic acid o taxifolin are not endogenous.

RE：Metabolite characterization was based on mass - MZ, RT and comparison of secondary fragments in the database.In the process of mass spectrometry comparison to the database, the exogenous substances may be characterized as the secondary debris with a higher grading value and a consistent retention time of mass and charge ratio, but it may also be other substances, which means that the mass and charge ratio and retention time can correspond to many substances. Therefore, we chose the secondary debris with a high matching degree.Therefore, there will be exogenous substances proposed by the reviewers.
